# Long-term corticosteroid use and dietary advice: a qualitative analysis of the difficulties encountered by patient

**DOI:** 10.1186/s12913-019-4052-y

**Published:** 2019-04-26

**Authors:** Muriel Nogué, Jacques Rambaud, Sylvie Fabre, Nathalie Filippi, Christian Jorgensen, Yves-Marie Pers

**Affiliations:** 1 Endocrinology therapeutic unit, Lapeyronie, University Hospital Lapeyronie, 371, avenue du doyen Gaston Giraud, 34295 Montpellier, France; 2 General Medicine Department, Montpellier, France; 3 Mutualist Hospital Beau-Soleil, Montpellier, France; 4 Clinical immunology and osteoarticular diseases therapeutic unit, Lapeyronie University Hospital, Montpellier, France

**Keywords:** Corticotherapy, Qualitative study, Therapeutic education, Lifestyle change, Diet

## Abstract

**Background:**

Nearly 1% of the population is currently treated with long-term corticosteroid therapy. When corticosteroids are introduced, information concerning potential adverse effects and recommendations for lifestyle changes aimed at preventing such effects is provided to patients. However, studies have shown patients often do not fully comprehend the information provided and have difficulty implementing the recommended dietary and physical activity advice. In this study, we aim to highlight the difficulties encountered by patients in comprehending and implementing recommendations in the context of long-term corticosteroid use. Such information can be used to better optimize care, particularly concerning adherence to the treatment, the diet, and thus improve the quality of life of patients.

**Methods:**

We recruited adult patients under long-term corticosteroid (≥ 3 months, ≥ 5 mg/day) treatment from both general medicine and rheumatology practices. We performed a qualitative analysis based on semi-structured interviews of these patients. Transcripts of these interviews were then compiled and analysed using a thematic approach.

**Results:**

Sixteen patients were included. Analysis of the interviews revealed that patients’ hope for effective corticosteroid treatment was counterbalanced by concerns over potential adverse effects. In some patients, the need to respect a strict and imposed diet induced psychological distress, potentially leading to eating disorders or fear of social exclusion. Furthermore, patient ambivalence toward the therapeutic education was highlighted, as well as the notion of filtering information, conscious or unconscious, as revealed by their lack of recall. The relationship with the physician also affected the treatment experience.

**Conclusion:**

Our analysis of the personal experience of patients regarding recommended lifestyle changes associated with long-term corticosteroid treatment highlights patient difficulties and suggests different ways of improving therapeutic education.

**Electronic supplementary material:**

The online version of this article (10.1186/s12913-019-4052-y) contains supplementary material, which is available to authorized users.

## Background

Globally, nearly 1% of the population is currently treated by long-term corticosteroid therapy [1–5], most notably in the treatment of rheumatologic, pneumologic, neurologic, and autoimmune conditions such as rheumatoid arthritis, asthma, Horton disease, and Crohn disease and ulcerative colitis, respectively. At the initiation of treatment, information about adverse events (AEs) and methods of prevention through diet and lifestyle changes is usually provided to patients [6]. Regarding osteoporosis, obtaining adequate supplies of calcium and vitamin D as well as promoting physical activity are recommended by the National Health Authority, and included in the information provided to patients [1]. Dietary recommendations include reducing salt, sugar, and caloric intake, while at the same time increasing the intake of protein and potassium. However, at present, there are currently no official recommendations, and due to the lack of evidence concerning the efficacy for these measures, patients may receive expert advice that could differ among clinics [7–9]. Several quantitative studies have assessed the AEs of long-term corticosteroid therapy and the variety of different regimens adopted by physicians to reduce the risk of their development [10–12]. Other studies have evaluated patient adherence to the treatment [13–15], their knowledge and perception of AEs [16–19], as well as patient commitment to following dietary advice and recommendations for physical activity [20, 21].

The purpose of our work is not to discuss the value of such measures, but to analyse self-reported difficulties that patients encounter in following the recommendations. Indeed, previous studies have also highlighted challenges patients experience in following hygiene or dietary advice [22] and imperfect acquisition of information provided by the health practitioner regarding the treatment and prevention of undesirable side effects [23]. Thus, physician advice meant to optimize care does not entirely fulfill the patient’s needs. These needs may be better met when, taking into consideration the beliefs and expectations of patients resulting in an improved therapeutic alliance and the adherence to treatment [24, 25].

We, therefore, carried out a qualitative study with patients undergoing long-term corticosteroid therapy to understand the difficulties they might encounter when implementing the lifestyle and dietary advice given by physicians.

## Methods

### Study population

We chose to carry out a qualitative study as a first step to better understanding patient difficulties in adhering to physician recommendations aimed to minimize AEs during long-term corticosterioid therapy. The study was carried out between April and August 2015, with patients receiving corticotherapy and being followed in a rheumatology or general medical practice. We included adult patients aged ≥18 years, taking corticosteroids at an average dose of ≥5 mg/day of equivalent prednisone, and who had been in corticosteroid treatment for ≥3 months. Exclusion criteria were major memory impairment or a language barrier. Patients were invited by their rheumatologist or GP to participate in the study. The study has been registered by CLER validating the study methodology (RECHMPL17_0330), and ethical approval obtained from the local council of ethics for research.

### Interviews

For each patient, a semi-structured interview was carried out. To obtain informed consent for participation in the study, oral and written information concerning the conduct of the trial and the right to withdraw from participation at any time was given to each patient. The same investigator, M.Nogue, used a guide (Additional file 1) designed according to the accepted criteria of qualitative methodology [26–28] to conduct all the interviews. The interview guide was based on the investigator’s fundamental knowledge of corticosteroid therapy, on an extensive literature review of adverse effects and patient difficulties in adhering to treatment, and from experience of questions frequently raised by patients and the expression of their difficulties during consultations in both rheumatology and general medicine. The most common questions asked by patients fell along three axes: treatment, treatment complications, and the received lifestyle advice. Moreover, five themes were identified to understand patient difficulties: the patient living environment; patient knowledge of corticosteroids; patient perception of the beneficial and undesirable effects of corticosteroids; health and dietary advice and their implementation; and finally, questions on how to improve care management.

Our interview guide is provided as Additional file 1. While we did not perform a specific pilot study to assess question relevance, we did implement some minor changes after the first three interviews to take into account particular issues on the formulation. Some questions were deemed to lack detail and were therefore changed in the guide to facilitate more thorough patient answers. For example, the question *“Since you’ve been taking cortisone, how do you feel?”* was followed up by two sub-questions *“What positive effects do you feel from the treatment, and what negative effects?”* We chose to build the guide with open-ended questions. For example, we proposed questions such as “Can you explain to me what the Corticosteroid treatment represents for you, and why you need to take it every day?” or “What did you think about the content of medical advice given, in regard to your expectations? What did you feel when you received this advice?”

At the end of the interviews, the entire recorded audio data was transcribed to facilitate thematic analysis by the primary investigator. The first stage of the analysis consisted of an initial cursory reading, to establish an overview of the data, followed by a more careful reading to highlight keywords and phrases. These keywords and phrases were then grouped by theme. An external qualitative researcher cross-referenced the emerging themes and keywords. The results were presented to the research team to verify the plausibility and exhaustiveness of findings and to allow, through data triangulation a high validity of the conclusions [26].

## Results

### Patient characteristics and interviews

Sixteen patients were included in the study: eight recruited from a rheumatology clinic and eight from a General Practitioner (GP). The main characteristics of the participants are summarized in Table 1. The patient population was mainly women (69%). Patients in the rheumatology group were slightly older than those recruited from the GP (62 years versus 52.6 years, respectively) although this age difference was not significant. Among the patients in the GP group, corticosteroids were prescribed for a variety of medical conditions, while all of the patients in the rheumatology group were followed for rheumatoid arthritis. The corticoid dosages and duration of the therapy were both higher in the rheumatology group (respectively 9.3 mg of equivalent Prednisone over 15.2 years and 7.6 mg over 3.9 years in the GP group) with a significant difference in the duration of corticosteroid use (*p* = 0.046).

**Table 1:** Patient characteristics

	Whole population *n* = 16	Rheumatology *n* = 8	General Practice *n* = 8	*p*
Women: n (%)	11 (68.7%)	6 (75%)	5 (62.5%)	NS
Age (years): mean (DS) [min- max]	57.3 (14.4) [23–83]	62.1 (12.9) [48–83]	52.6 (15.9) [23–70]	*p* = 0.362
Job status: n (%)				*p* = 0.412
- Retirement	7 (43.7%)	4 (50%)	3 (37.5%)	
- Active	6 (37.5%)	2 (25%)	4 (50%)	
- Disability	2 (12.5%)	2 (25%)	0	
- Student	1 (6.25%)	0	1 (12.5)	
Chronic diseases**:** n (%)				*p* = 0.007
- Rheumatoid arthritis	10 (62.5%)	8 (100%)	2 (25%)	
- Giant cell arteritis	2 (12.5%) 1	0	2 (25%)	
- Eosinophilia	(6.25%)	0	1 (12.5%)	
- Auto-immune hepatitis	1 (6.25%)	0	1 (12.5%)	
- Undifferentiated rheumatism	1 (6.25%)	0	1 (12.5%)	
- Giant urticaria	1 (6.25%)	0	1 (12.5%)	
Steroid duration (years): mean (ET) [min-max]	9.53 (10.21) [0.5–28]	15.2 (10.78) [0.5–28]	3.88 (5.85) [0.5–16]	*p* = 0.046
Steroid dose over the last 3 months (mg EP), mean (ET) [min-max]	8.37 (4.08) [5–17.5]	9.3 (3.76) [5–15]	7.4 (4.43) [5–17.5]	*p* = 0.119

*EP* Equivalent Prednisone

Interviews were conducted in a consultation room. In one case, the interview was conducted at the patient’s home due to restricted mobility. The average length of interviews was 54 min. They were performed until data saturation was reached, i.e., when the data collected ceased to provide any additional information on the issues contained in the interview guide [26].

### Patient views of steroid drugs and treatment

**Fig. 1:**
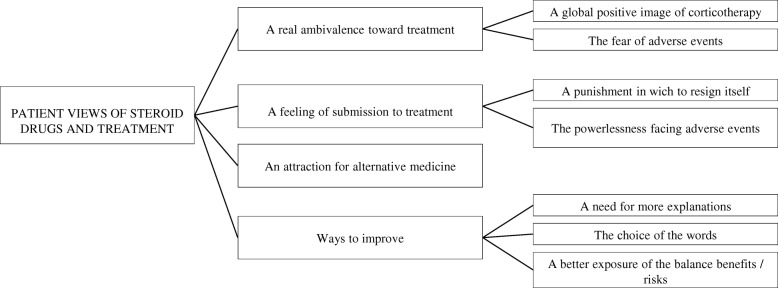
Patient views of steroid drugs and treatment

Many patients showed a significant ambivalence towards their corticotherapy (Fig. 1). Indeed, perceptions of treatment effectiveness and the benefits of corticoids were often counterbalanced by fears of its adverse effects. *«It is a very good drug, but with very perverse effects! » (I13).* Patients expressed feelings of submission to treatment, likened as a punishment to which they had to resign, and fatalistic views of corticotherapy’s side effects. *When I saw that I had no choice I told myself» «Good go ahead ... I give up, I take it…» «Because I could not do otherwise» (I2).* Some patients showed an interest in alternative medicines, which were perceived as milder. *«So I was looking for an alternative ... in soft medicine in parallel with my background treatment, if I could find…» (I2).* Finally, patients were asked about possible ways to improve their relationship with the treatment. More information about the drugs, avoiding frightening words in describing potential side effects and a better explanation of the benefit / risk balance were among the preferred options.

### Patient views of dietary advice

**Fig. 2:**
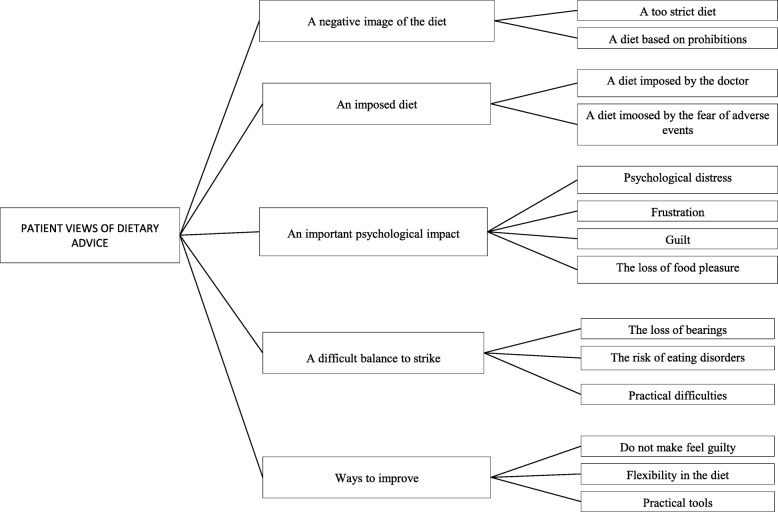
Patient views of dietary advice

The majority of patients reported dissatisfaction with the dietary advice (Fig. 2), which was perceived as too strict and often exacerbated fears of developing adverse side effects.*«It was stressful because, on the list of products that were allowed, I did not have much» «I think I had too many forbidden foods» (I3).* The dietary advice led to frustration, guilt, and loss of food pleasure, often with strong psychological repercussions. *«And then I will tell you, if you have no right to drink a shot, if you have no right to eat things, it is better to die.» (I14)* Patients reported often forgetting the basics of nutrition due to multiple and sometimes conflicting advice, and sometimes the emergence of eating disorders. *«There were some food frustrations and these deviations made me think to bulimia. I gorged myself with what I had no right» (*I6*).*

To improve this aspect, patients proposed allowing some flexibility in the diet, advice on reducing guilt, and the integration of practical tools, such as providing a list of bakeries that make salt-free bread.

### Patient views of recommended physical activities (PA)

**Fig. 3:**
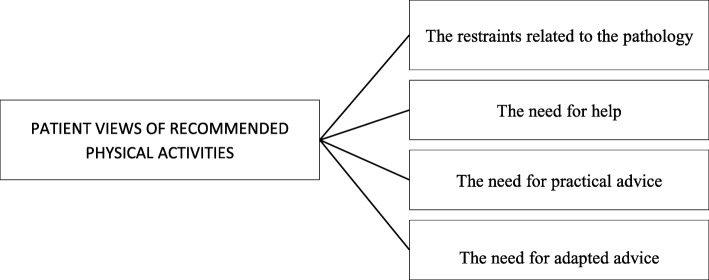
Patient views of recommended physical activities

Concerning the practice of PA, patients emphasized difficulties associated with background pathology (Fig. 3), especially those in the rheumatology group. *«But I would like to do something again this year, but not something like oriental dance, something more adapted to my disease*» (I16). To improve physical activity recommendations, patients proposed that physicians help in choosing activities and providing practical advice specially adapted to each patient.

### Patient views of information and education on steroid use

**Fig. 4:**
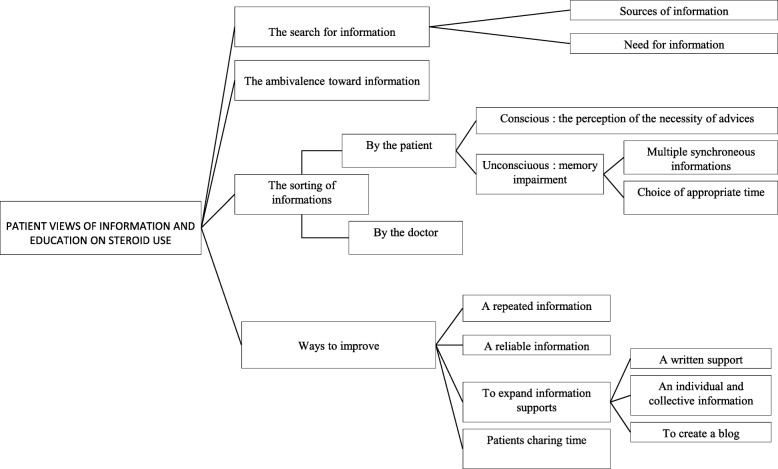
Patient view of information and education on steroid use

Despite having received information from their physician, many patients reported needing to seek additional information from a variety of sources (Fig. 4), including documents provided by medical and paramedical practitioners, websites, or television programs. *«How I knew it, I do not know, maybe watching Dr. House…»* (I5) *«Today even if you are not aware, you will be searching on the internet and you will learn lots of things.»* (I4) Patients consistently reported an ambivalence when facing information that they sometimes found disturbing. An interesting notion of filtering information emerged during the interviews, either conscious or unconscious, judging from the need for repeating advice and the apparent lack of recall*. «It’s true that the doctor gave me all the information, I’m sure, but I was not able to assimilate them right away» (I5)* Indeed, information regarding details of treatment, dietary restrictions and PA provided at the initiation of corticotherapy often comes directly on the heels of chronic pathology discovery. This can result in patients becoming overwhelmed, making it difficult to memorize all of the advice and information they are provided. Lastly, some information filtering by the physician was also described, perhaps due to a reluctance to address all the adverse effects of corticosteroids when treating an anxious patient. *«In addition, they feel that I am anxious about the treatment, which is somehow true, therefore they also purposely avoid to tell me things because they say after she won’t take anything but I want to know anyway» (I16).*

Patients stated that one way to improve how information on treatment and its adverse effects is provided would be by providing repeated, reliable, and individualized information with the help of a variety of supports that adequately address their different needs and expectations. Interest in a written notice was frequently proposed, but did not seem to be sufficient for most patients and should not substitute for the advice given by the physician. Other suggestions were also made, such as establishing a patient support group allowing for the expression of difficulties encountered during therapy and the sharing of practical tools.

### Patient relationships with the medical and paramedical practitioners

**Fig. 5:**
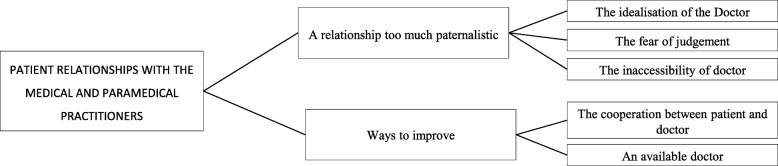
Patient relationships with medical and paramedical practitioners

The caregiver-patient relationship was sometimes described as very paternalistic (Fig. 5). *«Doctors, I find that often I disturb them asking too many questions. It bothers them because they feel a little put in... in doubt» (I16).* In this sense, patients believed that more cooperation and availability of the physician might improve care management.

### Patient relationships with their relatives

**Fig. 6:**
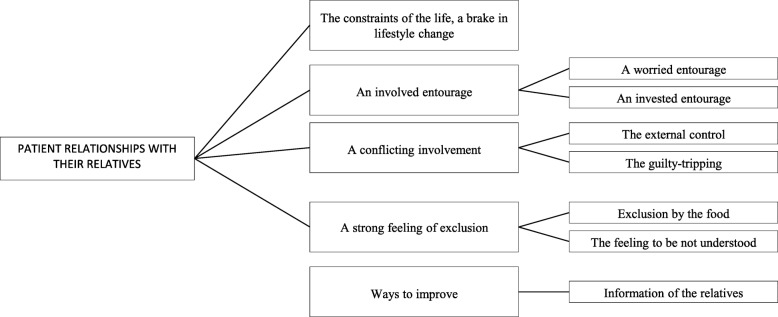
Patient relationships with relatives

This final topic emerged during the interviews. Relatives play an important role in the patient care experience and while sometimes helpful, they may also contribute to increasing patient difficulties (Fig. 6). Indeed, while families are described as willing and prone to help, in some cases even starting to follow the same dietary measures, this can sometimes become excessive. *«And then afterwards there are friends, family, who are also giving good advice too. Even this can be heavy. » (I10).* Patients often experienced familial involvement in a conflicted manner, with the help being perceived as a tool of external control and sometimes eliciting guilt, due to misunderstandings of the situation on both sides. These elements combined to lead to exclusion for a majority of the participants. *«I stopped eating with my husband actually, because I was too tempted» (I3).*

### Comparing interviews between specialist and GP patients

When comparing the interviews of patients from the rheumatology group with those of the GP, the results were substantially similar. However, it appeared that patients with a more flexible view of the dietary regime were more numerous in the GP group than those in the rheumatology group (50% vs. 25%). It may suggest less restricting diet advices in the GP group, with terms as *“no total restriction” (I7),* « T*he restriction is not for me, I have to be able to enjoy myself» (I10).* Moreover, this might be related to the lower average age of patients in the GP group since the patient-caregiver relationship is transitioning from year to year towards a closer partnership. Indeed, regarding adverse effects, patients in the older rheumatology group, voiced more concerns about the future and were more attentive to the prevention of complications arising from corticosteroid therapy. Similarly, the physician / patient relationship differed significantly according to age, with older patients more often reporting a paternalistic relationship with their physician accompanied by a greater sense of confidence in, and profound respect for, their advice.

## Discussion

While corticosteroid therapy has been in use for many years, steps to improve treatment adherence and acceptance appear still to be needed. Our study highlights a self-reported need of patients for information that is reliable, accessible, consistent, individually adapted, and neutral, that is well provided by the physician and possibly supplemented with access to other forms of support provided by the medical structure. The low GP participation rate for inclusion in the study (only 8 of the 150 patients contacted through their GP accepted to participate) could be considered a limit to the study’s conclusions [29, 30]. This likely results from GP physicians not prioritizing enrollment of their patients in the study due to their heavy administrative workload and by the fact that medical consultations are most of the time short and for acute problems. These constraints on the patient / physician relationship in the GP setting support the idea that a patient support group could augment care. For example, such groups would allow for the expression of difficulties encountered by the patients and the exchange of practical tools to face such difficulties [31]. While families may also provide support, providing relatives access to reliable information may be very important to avoid the exclusion reported by many patients.

We have chosen to encourage patient reflection around the experience corticosteroid treatment and to provide a constructive critique of the content and form of the information received regarding their care. Such information can be used to optimize care, facilitate improving the therapeutic alliance, and enhance the quality of life of the patients under corticotherapy. While most authors use quantitative studies to simplify study design and interpretation, the qualitative method chosen for this study is best adapted for discovering the individual experiences and difficulties encountered by patients during care, and thus provides a link between the basic science of quantitative studies and the expressed needs and difficulties expressed by patients [27, 28]. Our study procedures, including an in-depth description of the context, sample design, audio-taping transcription, saturation, and triangulation of the analysis have ensured the reliability of the findings in our setting.

Some vital considerations emerged at the end of the study. Several patients commented on the potential psychological impact of strict dietary recommendations, with three patients evoking the risk of developing eating disorders. Patients who were more comfortable in adopting dietary advice in our study were those who had successfully adapted the advice to their daily routine, perhaps with measures that were more flexible than the ones proposed. Medical authorities know the dangers of food restrictions as a source of frustration for patients [32, 33], and how difficult it is to respect them over the long term. It is for these reasons that physicians should adapt their approach to discussing dietary recommendations to each patient. Although no previous study has been conducted on this topic using the same methodology, the literature confirms some of our results. The subject of anxiety due to fear of adverse effects, the influence of the benefits/ risk balance, and the search for information have been investigated in many previously published studies [14, 34]. Various needs highlighted in our study have also been identified by the EULAR (European League Against Rheumatism) committee as significant points to be developed in the management of patients receiving corticosteroid therapy; in particular the importance of providing explanatory information, repeating information contained in recommendations and the balance of benefits / risks [35].

The search for information by the patient is also a common feature of all studies on corticosteroid therapy. Indeed, a qualitative study carried out on five thematic groups reinforces the idea that reluctance to corticosteroid therapy was partly explained by the poor information provided by the prescribing physician [36]. While 75% of the patients reported that the best source of treatment information was the specialist physician, another 25% admitted to seeking information in the media and 22% asked about cortisone use in their own family [23]. One study showed that among the information-related topics, nutrition came as second only after general information concerning the product [34].

Concerning information modalities, the notion of written support in addition to the oral information provided by the physician, emerged from our study as an important improvement, although not a sufficient support by itself. The diversification of supports is a theme which also appears in our study and has been previously described elsewhere [24]. More than basic medical knowledge, the participants highlighted a need to exchange practical advice and share their difficulties with people having the same experience. The suggestion of a patient support group is relevant and highlights needs that only patients could bring to light. To achieve a better benefit / risk ratio, the psychological impact and changes in everyday life as a consequence of strict dietary recommendations, such as low-salt and low-sugar, should be taken into account [11, 23] particularly since their effectiveness has not yet been scientifically proven [6, 8, 13].

Finally, social relationships between physician / patient and relatives / patients are also poorly understood but appear to have a special place among patient concerns. If physician / patient relationships cannot be the target of major intervention measures, the relationship with relatives can be significantly improved by informing families. In this sense, nutritional guides addressed to the elderly have been produced that facilitate the involvement of relatives [37]. This point is also an axis of work defined by EULAR in 2013 [35].

## Conclusion

The need for more information regarding patients on long-term corticosteroid therapy has been highlighted in our study. In their own words, patients have described needs for more reliable, accessible, repeated, individually-adapted, and neutral information. Different tools have been suggested, with a common emphasis on the importance of oral information given by the physician. The tools proposed are written information with unrestricted accessibility, even in the case of geographical remoteness. In this case, educational brochures or a web page (the most used information media) were suggested. Also, this information should be provided in a medical structure to maintain its reliability and neutrality. Finally, a treatment support group could complement the information provided by physicians, allowing patients to discuss their difficulties with others sharing the experience of receiving corticosteroid therapy and providing a forum for the exchange of practical tools improving the quality of life.

## Additional file

Additional file 1: Interview guide. (DOCX 494 kb)

## Abbreviations

AEs: Adverse Events
CLER: Local council of ethics for research
CNIL: National Commission on Computer Technology and Freedom
EULAR: European League Against Rheumatism
GP: General Practitioner
I: Interview
IQR: Interquartile Range
PA: Physical Activities
