# Supplementary material for: Long-term corticosteroid use and dietary advice: a qualitative analysis of the difficulties encountered by patient
Source: BMC Health Serv Res. 2019 Apr 26;19:255. doi: 10.1186/s12913-019-4052-y (PMC6486686; doi:10.1186/s12913-019-4052-y)
Supplement: Supplementary file 1 — Interview guide. (DOCX 494 kb) [file 12913_2019_4052_MOESM1_ESM.docx]

**INTERVIEW GUIDE**

If you agree, the interview will be recorded, but it remains completely confidential and anonymous.

**This is a list of themes that will be addressed:**

*(The questions presented here will not necessarily be asked in this way, but each overall theme will be addressed in a manner depending on the relationship established between the interviewer - respondent; the information in italics is indicative for the interviewer in order to be used as a follow-up in the discussion):*

**1. The patient, who is she/he? Patient environment and living environment**

*(Age, sex, family situation, employment, place of meals, socio-cultural level,) :*

First of all, a few questions to get to know you better:

- If you were to introduce yourself in a few words to a stranger, what would you say?

- Can you describe in a few words one of your typical day, from waking up to bedtime including activities, meals, leisure...

**2. What does he know? Medical context of corticosteroid therapy**

*(Pathology requiring corticotherapy, type of treatment, dosage, duration...).*

- You have a cortisone treatment, can you tell me what cortisone is for you, and why you take it daily?

- How did you get this information? *(Time, place, contact person, initiative...)*

**3. How does it feel ?- The effects of the treatment**

- Since you've been taking cortisone, how do you feel?

*(What positive effects could you feel from the treatment, what negative effects?)*

- Did you hear about these effects before you felt them? Have you heard about the side effects of cortisone?

*(When was it treatment? Who told you about this? Who requested the information? Have you heard of it from anyone other than this person? Supports used? )*

- What did you think of the content of the information in relation to your initial expectations?

*(What did you want to know? Was any information missing or too much?)*

- How did you feel after this information? (worry/reinsurance/neutral...)

**4. What does it do ? – Lifestyle advice and its implementation**

- What has taking cortisone changed in your life?

- And more precisely in terms of diet and physical activity?

- For what purpose did you implement these changes? *(Advice from doctors, friends, fear of side effects...)*
